# Intervention to Improve Well-Being, Nutrition, and Physical Activity in Adults: Experimental Study

**DOI:** 10.2196/47251

**Published:** 2024-10-15

**Authors:** Morghane Aubert, Céline Clavel, Christine Le Scanff, Jean-Claude Martin

**Affiliations:** 1 Laboratoire Interdisciplinaire des Sciences du Numérique Orsay France; 2 Laboratoire Complexité, Innovation & Activités Motrices et Sportives Université Paris-Saclay Orsay France; 3 Laboratoire Complexité, Innovation & Activités Motrices et Sportives Université d'Orléans Orléans France

**Keywords:** mindfulness, well-being, affects, nutrition, physical activity, intervention, lifestyle habits, mindfulness exercises

## Abstract

**Background:**

Mindfulness improves well-being, improves emotional regulation, reduces impulses to eat, and is linked to increased physical activity. Mindfulness interventions usually focus on 1 aspect but do not offer an approach to holistically improving lifestyle.

**Objective:**

This study aims to address this gap by designing and evaluating a holistic mindfulness intervention.

**Methods:**

Committing to a 12-week intervention with 2-hour sessions without knowing whether you will enjoy it can be a hindrance for someone completely unfamiliar with mindfulness. For this reason, we decided to design a mindfulness intervention with short sessions over a reduced number of weeks. The aim is to enable novices to discover different aspects of mindfulness while at the same time offering a satisfactory practice for people who are already practicing mindfulness. We designed and evaluated a web-based mindfulness intervention in 5 sessions of 5 to 10 minutes each on well-being, diet, and physical activity to support a healthier lifestyle. The first 2 sessions focus on formal mindfulness meditation to enable novices to discover mindfulness and its main principles. Then there are 2 sessions about food. The first session about food aims to develop a sense of satisfaction with the food we eat and to focus our attention on new sensations. The second session about food aims to develop the ability to resist the lure of unhealthy foods. Finally, there is a session on physical activity. The aim is to develop a particular awareness of the body during movement, to increase satisfaction with physical activity, and to develop regular exercise.

**Results:**

In total, 32 participants completed the intervention. After the intervention, we observed decreases in negative affect, anxiety, and emotional distress, and an increase in dispositional mindfulness. There was no effect on reported healthy eating habits and physical activity habits. Few participants repeated the exercises as recommended. The majority of our participants were new to mindfulness. The majority of our participants reported being satisfied with the different sessions. A few minor difficulties were mentioned, mainly related to the environment in which the participants carried out the sessions. Only 1 session was less satisfactory for one-third of the participants. The session on resistance to unhealthy foods was formulated too strictly and the idea of banning certain foods was a hindrance for one-third of the participants. A reformulation is needed.

**Conclusions:**

The mindfulness exercises were well accepted and promoted a state of mindfulness. It would be interesting to provide easier technical access to the exercises via a mobile app so that they can be repeated easily.

## Introduction

### Overview

Mindfulness is defined as consisting of 3 distinct aspects: dispositional mindfulness, which is a personality trait; a spiritual pathway that can be used to bring well-being; and finally a state that can be induced and developed through practice (which could also develop the dispositional mindfulness trait) [1]. The practice of mindfulness could support the development and maintenance of a healthy lifestyle by increasing psychological well-being, promoting more controlled and less impulsive eating, and increasing satisfaction with physical activity.

### Background

Engaging someone in mindful attention prevents them from developing an approach bias toward appealing food images (eg, pizza). Limiting this approach bias through the development of dispositional mindfulness could limit the consumption of potentially unhealthy foods [2].

Mindfulness-based interventions have been developed to reduce binge eating [3]. So-called mindful-eating programs teach participants to be fully present to the sensations of their body when eating so that they enjoy eating more, eat more healthily, and decrease the amount they eat.

The results of 20 cross-sectional studies suggest a positive relationship between dispositional mindfulness and physical activity practice [4]. The results of 20 interventional studies suggest a positive effect of a mindfulness intervention on physical activity, but not all interventions have this effect [4].

On the other hand, the emotions felt during a physical activity would have an impact on the continuation of this physical activity. The positive emotions felt during a session of physical activity would have an effect on the spacing of the session with the next one and on the duration of the next session [5]. The more positive emotions an individual feels during a sports session, the less time they will wait to do their next session and the longer it will take. The practice of mindfulness could have an impact on the practice of physical activity through its impact on emotions. Indeed, a functional magnetic resonance imaging study showed an impact of mindfulness practice on emotional regulation [6]. Similarly, a meta-analysis showed a positive effect of a single mindfulness induction on the regulation of negative emotions [7]. Furthermore, a study shows that during a 10-minute walk, participants who walked while listening to a mindfulness recording reported more positive emotions and more enjoyment of the walk than those not listening to the recording [8]. According to a cross-sectional study, satisfaction mediates the effect of mindfulness on physical activity [9].

Of 89 studies included in a literature review with brief mindfulness interventions, 74 studies tested a single induction ranging from less than 5 minutes to 25 minutes in duration [10]. In addition, 49 out of 89 studies used an audio recording for the intervention. Despite the heterogeneous results, this literature review showed that a brief intervention can have a positive effect on emotional regulation, anxiety, and cognitive functioning. A limitation of these different studies is that they do not measure the medium-term and the long-term effects of these short interventions.

These different elements show that mindfulness seems to be a suitable tool to accompany and support a balanced lifestyle. However, traveling to a specific place and time to practice mindfulness is not always easy. Further, one solution is to make the mindfulness intervention available on a website.

A meta-analysis lists 15 web-based mindfulness interventions, that is, without being face-to-face with an instructor [11]. The results show that this type of intervention is effective in decreasing depression, decreasing anxiety, decreasing stress, increasing well-being, and increasing dispositional mindfulness.

### Objectives

The first objective of our study is to design and test a web-based intervention that aims to combine psychological well-being, nutrition, and physical activity. To meet this first objective, the web-based exercises that we have designed, based on the literature, are mindfulness sessions oriented toward nutrition, the body, walking, and the development of self-esteem. During each session, participants have to watch a web-based video and follow the instructions provided in the video. The video consists of a landscape photography accompanied by an audio.

The second objective of our study is to test an intervention over several weeks with very short sessions spaced 1 week apart to observe the medium-term impact in addition to the immediate impact of each mindfulness session.

To meet these objectives, we have 3 hypotheses. Our first hypothesis (H1) is that there will be an improvement in well-being after the intervention compared to before the intervention.

- Hypothesis H1A: After the intervention, there will be an increase in the positive affect score compared to before the intervention.
- Hypothesis H1B: After the intervention there will be a decrease in negative affect, psychological distress, and anxiety scores compared to before the intervention.

Our second hypothesis (H2) is that participants’ level of dispositional mindfulness will be higher after the intervention compared to before.

Our third hypothesis (H3) is that after the intervention the participants will report a higher number of days with a healthy diet and a higher physical activity score compared to before the intervention.

## Methods

### Intervention Design

It was decided to create a 5-week intervention. This study took place in France. The aim was to allow participants to practice different exercises while keeping the program short enough to appeal to those new to mindfulness. Several elements were taken into consideration: discovering the broad outlines of mindfulness, including a section on diet and a section on physical activity.

The 5- to 10-minute audio recordings are exercises guided by the voice of an expert mindfulness meditation instructor. In all, there are 5 audio recordings of guided mindfulness exercises. The first 2 recordings are exercises often used with meditation novices (body scanning and breathing). The following exercises alternate between eating and physical activity. The recordings were created with inspiration from existing recordings (videos in French by Christophe André, a psychiatrist and mindfulness expert; meditations from the Petit Bambou [FEELVERYBIEN SAS] mobile app, the best-known meditation app in France with over 5 million downloads) and indications from researchers who have worked on mindfulness [12-15].

The first exercise is a body scanning exercise so that participants can become familiar with the notion of being fully present and listening to the sensations of their body. The second exercise focuses on breathing to continue the familiarization with mindfulness. The third exercise is an exercise in mindful eating. The exercise consists of eating a piece of fruit or vegetable while listening to inner sensations. The fourth exercise is to walk in mindfulness while being guided by the voice of the instructor to know which sensations to focus on. The last exercise consists of learning to resist the urge to eat. The participant has to think of a food that he or she does not allow himself or herself to eat but would like to eat, and has to follow the instructor’s instructions to observe what happens to him or her when he or she thinks of this food.

### Recruitment and Protocol

Recruitment was carried out via various mailing lists and word of mouth. For example, 1 mailing list was Relais d’Information sur les Sciences de la Cognition. Relais d’Information sur les Sciences de la Cognition is composed of people who are interested in cognitive science and want to participate in experiments. Registration on this mailing list is open to all. When a request for participation was received, a link to the first set of questionnaires (Mindful Attention Awareness Scale, 12-item General Health Questionnaire, State-Trait Anxiety Inventory, Positive and Negative Affect Schedule, International Physical Activity Questionnaire) was sent for the preintervention measurement of the variables under study.

Recruited participants completed the questionnaires on a website before the start of the intervention. They were also asked about their height and weight, and whether they had ever practiced mindfulness meditation. The order of presentation of the questionnaires was randomized.

**Figure 1:**
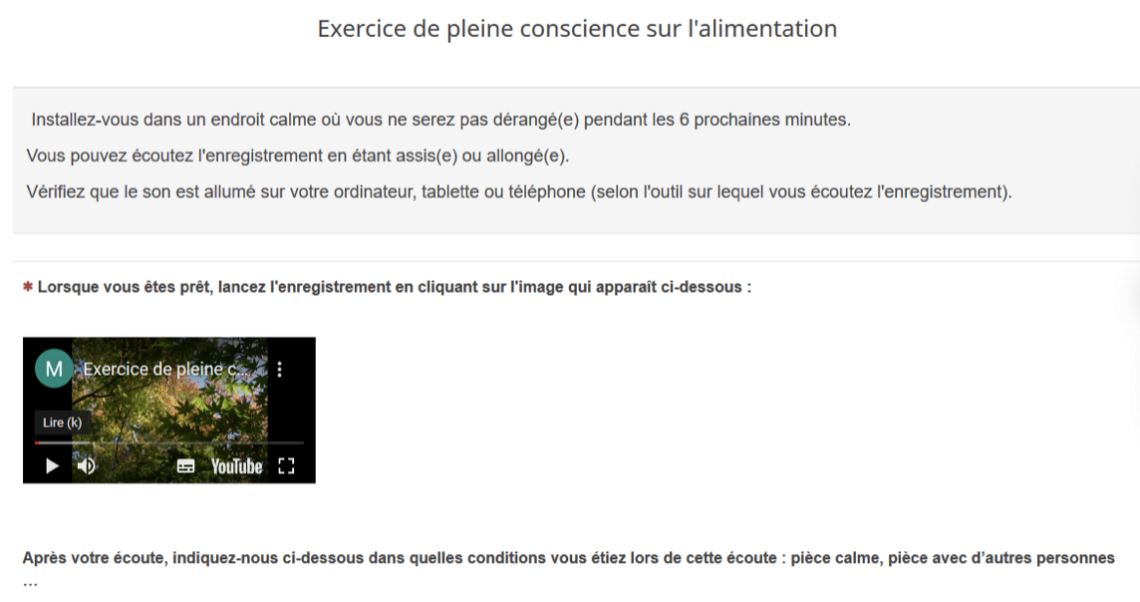
Screenshot of the fifth session. Translation: “Mindfulness exercise about food. Sit down in a quiet place where you won’t be disturbed for the next 6 minutes. You can listen to the recording while sitting or lying down. Check that the sound is switched on on your computer, tablet or phone (depending on the device you are listening to the recording on). When you’re ready, start the recording by clicking on the image below. After listening, tell us what conditions you were in when you listened: quiet room, room with other people, etc.”.

The first mindfulness exercise was sent to participants 2 to 7 days after they had completed the first questionnaire. The sending of subsequent exercises was conditional on the completion of this first exercise. A screenshot of the platform for the fifth session is shown in Figure 1.

We used the LimeSurvey platform for the first part of this study to collect information from the participants and their answers to the different questionnaires. For technical and experimental reasons, we continued to use LimeSurvey for the mindfulness intervention. For each session of the mindfulness intervention, participants had to access the LimeSurvey platform via a link they received by email. On each occasion, the first page presented the objective of the mindfulness session. On the next page, participants were taken to the mindfulness session. After the session, the participants had to answer questionnaires again.

After validating their answers with the postsession questionnaires, participants could no longer use the link provided. For this reason, they were given a permanent link to another platform to allow them to access the mindfulness sessions whenever they wanted to repeat the sessions. It was not possible to record data on this platform to check who was doing the exercises more than once. The number of repetitions of the exercises was determined solely by self-reporting by the participants.

In the instructions, participants were encouraged to listen to the recording at least three times a week and to listen to it whenever they felt the need. These recordings usually required participants to do something while listening to the recording (eg, eat a piece of fruit or vegetable). After each exercise, participants were asked several questions: satisfaction with the exercise, difficulties encountered, a French version of the Sport Motivation Scale for measuring mindfulness, the number of times they had listened to the exercise in the previous week (except for the first exercise), and their intention to repeat the new exercise. For the exercises relating to diet or physical activity, participants were asked about their intention to change their habits in this respect. Finally, participants were asked whether they would like to receive the next exercise.

When a participant completed an exercise for the first time via LimeSurvey, the research team received a validation email. The link to the next exercise was emailed 1 week later. If an exercise was not completed, participants received a reminder by email 3 to 4 days after receiving the exercise. If not completed, 1 week after this reminder, they received the next exercise with a reminder of the exercise not completed (except for the first exercise).

The 1-week interval between sessions was determined to give participants time to redo the exercises. A duration that was not too long was chosen to limit the risk of participants dropping out if they did not repeat the exercises between 2 sessions.

Additionally, 1 week after completing the last exercise, participants received a link to the second set of questionnaires, which included the same questionnaires as in the baseline measurement (Mindful Attention Awareness Scale, 12-item General Health Questionnaire, State-Trait Anxiety Inventory, Positive and Negative Affect Schedule, International Physical Activity Questionnaire). The order of presentation of the questionnaires was randomized.

**Figure 2:**
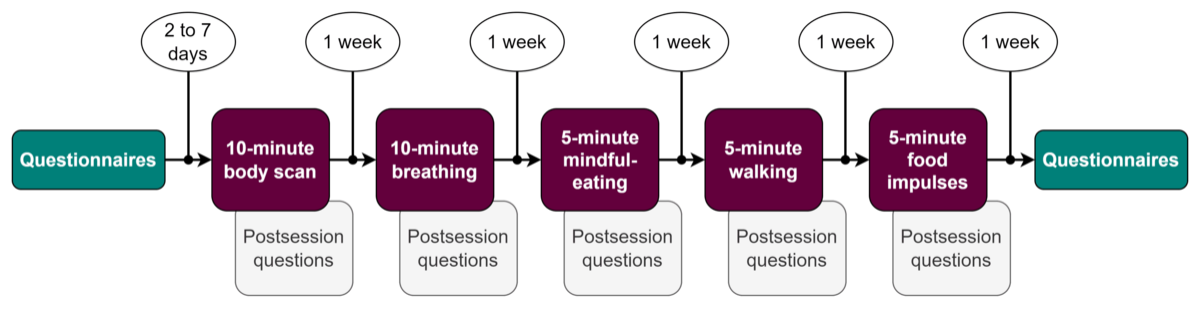
Course of the 5-week intervention (1 mindfulness session per week).

In total, 32 participants completed the entire intervention and the final questionnaires (Figure 2). Only these participants were included in the analyses. For each participant, their participation in a session was verified by their completion of the postsession questionnaires and the time spent on the session.

Participants did not have access to their answers unless specifically requested (which did not happen).

### Participants

Demographic data are presented in Table 1. The sample of 32 participants included 20 (63%) women. The average age was 44.4 (SD 15.9) years. In total, 30 (94%) participants did graduate studies. Further, 6 (19%) participants did undergraduate studies. Furthermore, 12 (38%) participants have a graduate degree. Additionally, 12 (38%) participants have a doctorate. We have no information on the employment of our participants. Moreover, 24 (75%) participants had no experience of mindfulness. Of note, 23 (72%) participants had a more or less regular practice that had some similarity to mindfulness (eg, yoga, tai chi, relaxation).

**Table 1:** Demographic data and baseline data values.

			Values
Gender (women), n (%)			20 (63)
Age (years), mean (SD)			44.4 (15.9)
**STAI^a^, mean (SD)**			
	Women	47.8 (8.1)	
	Men	40 (10.5)	
GHQ-12^b^, mean (SD)			12.8 (5.3)
PANAS^c^ positive, mean (SD)			32 (7.4)
PANAS negative, mean (SD)			17.6 (6)
MAAS^d^, mean (SD)			55.5 (12.4)
IPAQ^e^ METS^f^/week, mean (SD)			3640.9 (3428)
Healthy eating over 7 days (days), mean (SD)			4.6 (1.3)

^a^STAI: State-Trait Anxiety Inventory.

^b^GHQ-12: 12-item General Health Questionnaire.

^c^PANAS: Positive and Negative Affect Schedule.

^d^MAAS: Mindful Attention Awareness Scale.

^e^IPAQ: International Physical Activity Questionnaire.

^f^METS: metabolic equivalent of a task.

### Material

#### Measuring Mindfulness

A measurement of state mindfulness after each session was used to validate each exercise. A measurement of dispositional mindfulness before and after the intervention was used to observe whether there is a medium-term impact of the exercises.

#### State Mindfulness Scale

We translated the State Mindfulness Scale into French [16]. This scale measures the degree to which an individual is in a state of mindfulness at the time the questionnaire is completed. The score varies from 1 (not at all in a state of mindfulness) to 7 (completely in a state of mindfulness).

#### Mindful Attention Awareness Scale

Dispositional mindfulness was measured with the Mindful Attention Awareness Scale [17]. This is a 15-item scale. The score ranges from 15 to 90. The Mindful Attention Awareness Scale measures different aspects of mindfulness, such as attention to the present moment and nonjudgmental observation of feelings.

#### Measuring Well-Being

To assess the participants’ well-being, 3 questionnaires were used to measure both positive and negative aspects.

#### 12-Item General Health Questionnaire

The 12-item General Health Questionnaire is a scale to assess the psychological distress of an individual [18]. The score ranges from 0 to 36.

#### Positive Affect and Negative Affect Schedule

The Positive Affect and Negative Affect Schedule is composed of 20 items, half of which correspond to positive affect and the other half to negative affect [19]. The positive affect score ranges from 10 to 50. The negative affect score ranges from 10 to 50.

#### State-Trait Anxiety Inventory

The State-Trait Anxiety Inventory assesses the degree of anxiety in 20 items [20]. The score ranges from 0 to 80.

#### Behavioral Measures of Diet and Physical Activity

In total, 2 questionnaires are used to assess reported physical activity and eating habits.

#### International Physical Activity Questionnaire

The International Physical Activity Questionnaire assesses the amount of physical activity performed by an individual in the last 7 days in the metabolic equivalent of a task (METS)/week [21].

#### Dietary Habits

We adapted 5 items to measure the quality of eating habits [22]. The objective of these items is to observe whether there is a change in the number of days with a balanced diet between before and after intervention (eg, “In the last 7 days, indicate the number of days you ate a healthy, balanced diet”; reversed item: “In the last 7 days, indicate the number of days you ate high-fat products, such as red meat or high-fat dairy products”). The 5 items were averaged. The score ranges from 0 to 7 to reflect the number of days with a balanced diet.

### Statistical Analyses

Analyses were performed with R (version 4.0.2; R Foundation for Statistical Computing) software. Repeated measures ANOVAs were performed to test the different hypotheses. In addition to these tests, a repeated measures ANOVA was performed on the 5 state mindfulness scores between the 5 exercises.

### Ethical Considerations

This study was approved by a Research Ethics Committee (Comité d’Ethique de la Recherche) of Paris Saclay University (237). Consent was obtained from participants by a web-based validation on a written form. Data are anonymized and stored on a protected server.

## Results

### Overview

The results are presented in Table 2.

**Table 2:** Comparison baseline and postintervention values.

	Baseline, mean (SD)	Postintervention, mean (SD)	*F* test (*df*; ANOVA)	*P* value
MAAS^a^	55.5 (12.4)	59.9 (11.7)	5.5 (1,31)	.03
STAI^b^	44.8 (9.7)	40.9 (9.9)	12.8 (1,31)	.001
GHQ-12^c^	12.8 (5.3)	8.8 (4.1)	22.6 (1,31)	.003
PANAS^d^ positive	32 (7.4)	32.5 (9.5)	0.16 (1,31)	.69
PANAS negative	17.6 (6)	16 (5.2)	5.3 (1,31)	.03
IPAQ^e^ (METS^f^/week)	3640.9 (3428)	3896.7 (2970.8)	0.5 (1,31)	.49
Healthy eating over 7 days (days)	4.6 (1.3)	4.8 (1.1)	2.52 (1,31)	.12

^a^MAAS: Mindful Attention Awareness Scale.

^b^STAI: State-Trait Anxiety Inventory.

^c^GHQ-12: 12-item General Health Questionnaire.

^d^PANAS: Positive and Negative Affect Schedule.

^e^IPAQ: International Physical Activity Questionnaire.

^f^METS: metabolic equivalent of a task.

### Mindfulness State

**Figure 3:**
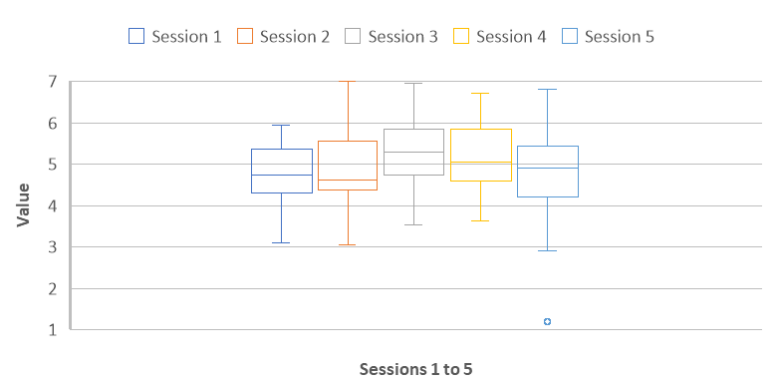
Mindfulness state.

A repeated measures ANOVA was performed on the mindfulness state measure. This value was statistically different between the 5 sessions (*F*_3,79_=3.82; *P*=.02; generalized η^2^=0.06). Bonferroni post hoc analyses for repeated measures showed that there were only 2 differences between the 5 sessions. The mindfulness state measured just after session 3 (mean 5.3, SD 0.8) was higher than the mindfulness state measured just after session 1 (mean 4.8, SD 0.7; *P*=.005), and it was higher than the mindfulness state measured just after session 2 (mean 4.9, SD 0.8; *P*=.003). The state of mindfulness measured just after session 4 (mean 5.2, SD 0.8) and that measured just after session 5 (mean 4.8, SD 1.1) did not differ from the mindfulness of the other sessions. The averages between sessions ranged from 4.8 (SD 1.1) to 5.3 (SD 0.8) on a scale of 1 to 7 (Figure 3). This result validates that the participants were, on average, in a state of mindfulness after the different exercises and that the exercises were well designed.

### Affects, Psychological Distress, and Anxiety

The level of anxiety in our sample appears to be slightly higher than that observed in the literature [23]. The level of psychological distress and negative affect in our sample appears to be somewhat lower than observed in the literature [21,24]. The level of positive affect in our sample seems similar to that observed in the literature [8].

A repeated measures ANOVA was conducted on the measure of positive affect between preintervention and postintervention. The preintervention measure of positive affect (mean 32, SD 7.4) did not differ from the postintervention measure of positive affect (mean 32.5, SD 9.5), *F*_1,31_=0.16, *P*=.69.

Hypothesis H1A was not validated.

A repeated measures ANOVA was conducted on the measure of negative affect between preintervention and postintervention. The negative affect measure was statistically different between preintervention and postintervention (*F*_1,31_=5.3; *P*=.03; generalized η^2^=0.02). The negative affect score decreased after the intervention (mean 16, SD 5.2) compared to before the intervention (mean 17.6, SD 6). Bonferroni post hoc analysis for repeated measures showed that this difference remained significant when adjusted with *P*=.03.

A repeated measures ANOVA was conducted on the measure of psychological distress between preintervention and postintervention. This measure was statistically different between preintervention and postintervention (*F*_1,31_=22.6; *P<*.001; generalized η^2^=0.15). The psychological distress score decreased after the intervention (mean 8.8, SD 4.1) compared to before the intervention (mean 12.8, SD 5.3). Bonferroni post hoc analysis for repeated measures showed that this difference remained significant when adjusted with *P*=.003.

A repeated measures ANOVA was conducted on the measure of anxiety between preintervention and postintervention. This measure was statistically different between preintervention and postintervention (*F*_1,31_=12.8; *P*=.001; generalized η^2^=0.04). The anxiety score decreased after the intervention (mean 40.9, SD 9.9) compared to before the intervention (mean 44.8, SD 9.7). Bonferroni post hoc analysis for repeated measures showed that this difference remained significant when adjusted with *P*=.001.

Hypothesis H1B was validated.

### Dispositional Mindfulness

The average preintervention dispositional mindfulness of our sample was similar to that obtained in the literature [25].

A repeated measures ANOVA was performed on the dispositional mindfulness measure between preintervention and postintervention. The dispositional mindfulness measure was statistically different between preintervention and postintervention (*F*_1,31_=5.5; *P*=.03; generalized η^2^=0.03). The dispositional mindfulness score increased after the intervention (mean 59.9, SD 11.7) compared to before the intervention (mean 55.5, SD 12.4). Bonferroni post hoc analysis for repeated measures showed that this difference remained significant when adjusted with *P*=.03.

The H2 hypothesis was validated.

### Behavioral Measures of Diet and Physical Activity

A repeated measures ANOVA was conducted on the measure of reported eating habits between preintervention and postintervention. This preintervention measure (mean 4.6, SD 1.3 days) did not differ from the postintervention measure (mean 4.8, SD 1.1 days; *F*_1,31_=2.52; *P*=.12).

A repeated measures ANOVA was performed on the measure of reported physical activity patterns between preintervention and postintervention. This preintervention measure (mean 3640.9, SD 3428 METS/week) did not differ from the postintervention measure (mean 3896.7, SD 2970.8 METS/week; *F*_1,31_=0.5; *P*=.49).

Hypothesis H3 was not validated.

### Acceptability of the Intervention

A summary of the acceptability data is presented in Table 3. Of the 65 people who completed the first exercise, 35 (54%) people indicated that they were very satisfied with the exercise, and 23 (35%) people indicated that they had difficulties with the exercise (staying focused, keeping up with the pace, etc).

**Table 3:** Session acceptability.

	Size sample, n	Satisfied and very satisfied, n (%)	Difficulties, n (%)
Session 1	65	35 (54)	23 (35)
Session 2	57	38 (67)	13 (23)
Session 3	46	31 (67)	6 (13)
Session 4	41	33 (81)	8 (20)
Session 5	36	19 (53)	14 (39)

Of the 57 people who completed the second exercise, 38 (67%) indicated that they were very satisfied with the exercise and 13 (23%) indicated that they had difficulties (especially with concentration).

Of the 46 people who completed the third exercise, 31 (67%) indicated that they were satisfied or very satisfied and 6 (13%) indicated that they had difficulties. Some of the chosen foods were less suitable for the exercise because they were not very tasty or difficult to keep in the mouth for a long time (eg, cucumber).

Of the 41 people who completed the fourth exercise, 33 (81%) indicated that they were satisfied or very satisfied and 8 (20%) indicated that they had experienced difficulties with the exercise. The reasons for this were varied: unsuitable space for a walking exercise, lack of concentration, too fast exercise without a quiet period before walking to focus on the present moment. Only 1 person indicated a certain rejection of the exercise because she did not see the point of the different exercises and found it unpleasant to hear phrases that were not addressed to her because the instructions to enjoy the sensations felt were in contradiction with the muscular pain felt by this person.

Of the 36 people who completed the last exercise, 19 (53%) indicated that they were satisfied or very satisfied and 14 (39%) indicated that they had encountered difficulties, mainly with the instruction to think of a forbidden food.

## Discussion

### Principal Findings

This study aimed to observe the impact of a web-based, short-term mindfulness intervention on psychological well-being, eating habits, and physical activity patterns.

To validate the 5 mindfulness-based exercises, a questionnaire measuring state mindfulness was completed by participants after each session. The results showed that the exercises induced a state of mindfulness with an average of 5 out of 7 (SD 0.2) for the 5 sessions. Overall, the level of mindfulness was stable between the 5 sessions. This result is consistent with the results obtained in the literature [26].

The fifth exercise, which dealt with food impulses, was problematic for just over one-third of the participants as it asked them to think about a food that they would not allow themselves to eat (without specifying a reason for this prohibition). In the free text box following the exercise, 12 (37.5%) participants indicated that they could not think of such a food because they do not prohibit themselves from eating any food. Several indicated that they did not find the idea of banning foods healthy. The wording of this exercise should be reviewed to soften the notion of prohibition, which does not correspond to everyone’s experience. The interest of such an exercise was to facilitate resistance to foods that are not recommended for health. Indeed, according to a study, imagining oneself in a situation of refusing chocolate leads to a less positive evaluation of chocolate and favors the resistance to the food impulse that could be present toward chocolate [27]. A possible reformulation would be to direct thoughts toward a sweet or fatty food without presenting it as forbidden, but more as a food not recommended for health.

The results of our study do not show an increase in positive affect after the intervention but they do show a decrease in negative affect, anxiety, and emotional distress between preintervention and postintervention. In a study, participants listening to mindfulness audio during a 10-minute walk reported more positive affect than those not listening to mindfulness audio [8]. However, this difference in positive affect was only observed during the exercise. Further, 5 minutes after the exercise, the level of positive affect of participants was similar between the 2 groups. In another study, a decrease in positive affect was observed after an intervention of 4 mindfulness sessions [28]. Our study increased participants’ psychological well-being through a decrease in participants’ negative feelings but not an increase in their positive feelings.

Our intervention did not have an impact on reported healthy eating habits and physical activity patterns. It is possible that the participants recruited did not need any help regarding nutrition and physical activity but rather for emotional distress and negative affect. This would explain why the results show a greater impact of the intervention on aspects related to emotional distress and negative affect than on the other aspects. It is also possible that the number of mindfulness sessions was not sufficient to observe an effect on these elements. Indeed, there was only 1 session on physical activity and 2 sessions on eating. It is also worth noting that for more than one-third of the participants, the second exercise on food was not relevant because of the wording about not eating certain foods.

A significant number of participants dropped out of the ongoing intervention. We had 37 (54%) participants who completed the first questionnaire without participating in the full intervention. In a meta-analysis of the effectiveness of mindfulness interventions delivered on websites, 5 studies assessed participants’ adherence based on their participation in the full sessions offered. In these studies, adherence ranged from 40% to 92% [10]. Our adherence percentage of 46% is in line with these results. Our intervention was carried out by email and links to web-based questionnaires and videos. In the meta-analysis, interventions were delivered via a website, web-based classroom, or phone apps [10]. It seems that accessing the exercises in our intervention was not instinctive. Indeed, the link we provided to participants by email allowed them to do the exercise only once. Another link was provided at the end of each session for participants wishing to repeat the exercise. Despite the encouragement to repeat each exercise several times, the self-reported responses indicate that few exercises were indeed repeated. Several participants reported difficulties in finding the recording because the link provided at the end of each session was not copied and could not be accessed afterward. Although this was specified in the instructions, it made access more difficult than if the intervention had been embedded in a full-fledged website or app.

### Limitations

The design of the intervention did not allow for verification that participants were performing the mindfulness exercises. It was also not possible to check the number of times the exercise was repeated. The 5-week intervention may not be long enough to bring about changes in behavior. It should also be noted that the sessions on diet and physical activity were held in the final weeks of the intervention. Longer-term follow-up should be considered in future studies and could provide additional information on the impact of the intervention. Another limitation is that our participants may not have accurate knowledge of health recommendations for diet and physical activity. It would be useful to combine our intervention with information on this subject. Finally, there was no objective measurement of dietary behavior and physical activity patterns.

### Conclusion and Future Directions

Our study suggests that a short-term web-based mindfulness intervention improves psychological well-being through a reduction in negative affect, anxiety, and emotional distress. The intervention also increased the participants’ level of dispositional mindfulness.

This type of intervention allows for low-cost, large-scale, everyday support.

Access to the exercises could be nevertheless facilitated by using a website with the exercises directly accessible and an individual connection of the participants which would allow a more precise follow-up. The advantage of a website or a mobile app is that it could also include information on health recommendations that are easily accessible and linked to the proposed exercises. Another advantage of a website or a mobile app to explore in future studies would be to leave the choice of exercises to the participants to encourage a feeling of autonomy and thus promote commitment and reduce the drop-out rate.

To go beyond reported intentions in the questionnaires related to physical activity and nutrition, it is necessary to include an objective measure of the behavior with, for example, a measure of the number of steps.

## Abbreviations

METS: metabolic equivalent of a task

## Data Availability

The dataset is available on Open Science Framework [29].
